# Minimal Internal Radiation Exposure in Residents Living South of the Fukushima Daiichi Nuclear Power Plant Disaster

**DOI:** 10.1371/journal.pone.0140482

**Published:** 2015-10-20

**Authors:** Junichi Akiyama, Shigeaki Kato, Masaharu Tsubokura, Jinichi Mori, Tetsuya Tanimoto, Koichiro Abe, Shuji Sakai, Ryugo Hayano, Michio Tokiwa, Hiroaki Shimmura

**Affiliations:** 1 Jyoban Hospital, Tokiwa Foundation, Iwaki, Fukushima, Japan; 2 Department of Radiation Protection, Soma Central Hospital, Soma, Fukushima, Japan; 3 Division of Social Communication System for Advanced Clinical Research, Institute of Medical Science, University of Tokyo, Minato-ku, Tokyo, Japan; 4 Department of Radiation Protection, Minamisoma Municipal General Hospital, Minamisoma, Fukushima, Japan; 5 Department of Diagnostic Imaging and Nuclear Medicine, Tokyo Women's Medical University, Shinjuku-ku, Tokyo, Japan; 6 Department of Physics, The University of Tokyo, Bunkyo-ku, Tokyo, Japan; University of South Carolina, UNITED STATES

## Abstract

Following the Fukushima nuclear power plant disaster, assessment of internal radiation exposure was indispensable to predict radiation-related health threats to residents of neighboring areas. Although many evaluations of internal radiation in residents living north and west of the crippled Fukushima nuclear power plant are available, there is little information on residents living in areas south of the plant, which were similarly affected by radio-contamination from the disaster. To assess the internal radio-contamination in residents living in affected areas to the south of the plant or who were evacuated into Iwaki city, a whole body counter (WBC) screening program of internal radio-contamination was performed on visitors to the Jyoban hospital in Iwaki city, which experienced less contamination than southern areas adjacent to the nuclear plant. The study included 9,206 volunteer subjects, of whom 6,446 were schoolchildren aged 4–15 years. Measurements began one year after the incident and were carried out over the course of two years. Early in the screening period only two schoolchildren showed Cs-137 levels that were over the detection limit (250 Bq/body), although their Cs-134 levels were below the detection limit (220 Bq/body). Among the 2,760 adults tested, 35 (1.3%) had detectable internal radio-contamination, but only for Cs-137 (range: 250 Bq/body to 859 Bq/body), and not Cs-134. Of these 35 subjects, nearly all (34/35) showed elevated Cs-137 levels only during the first year of the screening. With the exception of potassium 40, no other radionuclides were detected during the screening period. The maximum annual effective dose calculated from the detected Cs-137 levels was 0.029 and 0.028 mSv/year for the schoolchildren and adults, respectively, which is far below the 1 mSv/year limit set by the government of Japan. Although the data for radiation exposure during the most critical first year after the incident are unavailable due to a lack of systemic measurements, the present results suggest that internal radio-contamination levels more than one year after the incident were minimal for residents living south of the crippled Fukushima nuclear plant, and that the annual additional effective doses derived from internal Cs contamination were negligible. Thus, internal radio-contamination of residents living in southern radio-contaminated areas appears to be generally well controlled.

## Introduction

Following the Great East Japan Earthquake (GEJE) on March 11, 2011, various health threats have emerged at different levels and times [1,2]. Immediately after the disaster, health care systems serving local residents were seriously affected due to infrastructure damage and the shortage of medical materials [3,4]. Even after recovering from the earthquake damage, a number of residents in the affected areas continued to suffer negative effects, particularly in terms of mental health [5,6]. Among the areas affected by the GEJE, regions surrounding the Fukushima Daiichi nuclear power plant commanded more careful attention to health threats, since these regions were exposed to large amounts of radionuclides that were released from the crippled nuclear plant [7,8]. In general, excess radiation exposure is considered to be a risk factor for increased tumor incidence and tumor development [9]. As such, accurate assessment of radiation exposure of residents in the radio-contaminated areas is effective in encouraging the residents to be aware of both acute and chronic health problems that can be caused by radiation exposure [10,11]. For this purpose, the air dose rates in the affected areas were monitored to prevent unnecessary external radiation exposure for the residents. Although several zones remain evacuated, residents may now return to areas with lower levels of contamination [12]. Through such systematic and careful measurement of the air dose rate in the entire affected area, the acute health risk caused by the external radiation exposure appears to be lowered for residents evacuated from areas with contamination that exceeded safe limits for radiation exposure doses [13,14]. In contrast to external radiation exposure, exposure to internal radiation for each resident in the contaminated areas may be quite variable, since internal radio-contamination arises from intake of contaminated food and water, and thus depends on personal lifestyles [15]. To assess health risks caused by excess radiation exposure from radionuclide contamination, individual measurement of internal radio-contamination is indispensable.

Soon after the earthquake, local municipalities in the Fukushima prefecture began evaluating the internal radiation exposure of each resident by means of a whole body counter (WBC). In the local municipality Minamisoma, which is about 20 km north of the Fukushima nuclear plant, monitoring of internal radiation exposure to released radioactive Cesium (Cs) began 4 months (July 2011) after the nuclear plant incident [16]. A six-month survey (September 2011 to March 2012) that enrolled 9,498 residents, of whom 1,432 were children (6–15 years), found that one third of the residents had detectable levels of internal Cs contamination [17]. However, except for one resident (1.07 mSv/year), the effective Cs doses of these residents were lower than the government-allowed limit (1 mSv/year). Moreover, monthly assessment of the children in the study revealed that the detection rate among the tested children rapidly decreased within 6 months (from 57.5% to less than 2%) with very low levels of sustained internal radio-contamination [18], and after 7 months all of the tested children had undetectable internal levels of radioactive Cs [19]. These early studies indicate that careful monitoring of contaminated food intake and regulated inspection and distribution of commercial foodstuffs is likely sufficient to prevent residents from consuming radio-contaminated food. Thus, monitoring of internal radiation exposure by WBC is clearly effective in encouraging the residents to avoid further internal radio-contamination, and can reduce health risks. However, another study found that a small number of residents (9/30,622 study participants) exhibited relatively high levels of internal radio-contamination (> 50 Bq/kg Cs-137, equaling ~0.1 mSv/year). Subsequent evaluation and dietary counseling revealed that these residents continued to consume locally grown produce and meats from non-domesticated animals that had not undergone radiation inspection [20]. Dietary intervention measures that urged residents with high levels of internal contamination to consume only inspected food was quite successful for eight of the nine subjects who showed high internal radio-contamination, which was reduced by half after 3 months. This three-month period corresponds to the biological half-life of Cs.

Compared to the systematic WBC monitoring in areas north of the Fukushima Daiichi nuclear power plant, there is less information on internal radio-contamination for residents living south of the plant [21], even though the radio-contamination in this region was similar to the northern areas. In this study we surveyed residents living south of the nuclear power plant to determine whether their internal radio-contamination levels were similar to their northern counterparts. Similar to residents living north of the nuclear power plant, we detected internal radio-contamination in residents living to the south of the plant one year after the incident, but their levels were also minimal and within the safety limit (1mSv/year). Furthermore, within three years of the earthquake these elevated levels gradually decreased to undetectable levels for all but one study subject. Taken together, these findings suggest that internal radio-contamination of residents in radio-contaminated areas both north and south of the plant is minimal and generally well controlled.

## Materials and Methods

**Fig 1:**
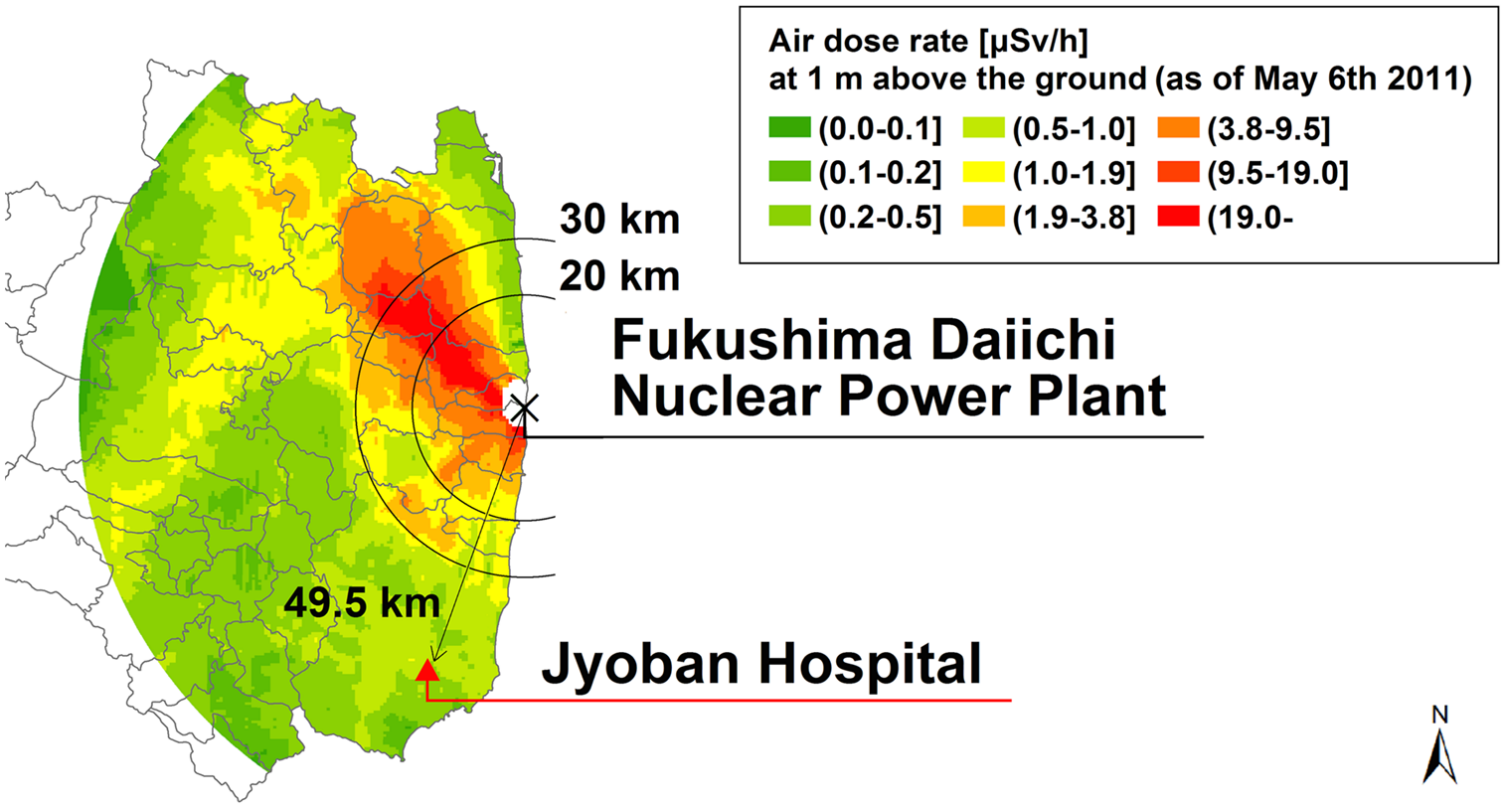
Areas to the south of the Fukushima Daiichi nuclear power plant. WBC measurement was performed at the Jyoban Hospital in central Iwaki, which is located about 50 km south of the Fukushima Daiichi nuclear power plant. Study subjects were mainly hospital visitors from Iwaki and its suburbs.

An internal radiation exposure-screening program was conducted between April 1^st^, 2012 and December 31^st^, 2014 to assess internal radio-contamination among residents in the affected southern areas. Study subjects were among voluntary visitors to Jyoban Hospital in Iwaki city (Fig 1) and were examined after obtaining informed consent to collect data on age, sex, and results of the internal radiation exposure-screening program during the study period. We also calculated the annual effective doses from internal Cs-137 contamination.

### Internal radiation exposure screening program

WBC measurements were conducted at the Jyoban Hospital between April 1, 2012 and December 31, 2014. Major variables recorded for this study were age, sex, and the total body burden of radioactive Cs (Cs-134 and Cs-137), which represented the total internal radiation exposure from radionuclides dispersed from the crippled nuclear power plant. Internal Cs contamination was measured as total body contamination. Prior to the WBC, study subjects changed into a hospital gown so that radio-contamination from their clothes was not detected.

### Test procedure

The WBC device used for this study was a stereoscopic apparatus with two 3 x 5 x 16 in^3^ NaI scintillation detectors (Fastscan Model 2251; Canberra, Inc., Meriden, CT, USA). Schoolchildren shorter than 130 cm stood on a 30-cm stool during the measurement. For a two-minute scan the Cs detection limits were 220 and 250 Bq/body for Cs-134 and Cs-137, respectively.

### Effective dose calculation from Cs-137 internal contamination

Annual effective doses from internal Cs-137 contamination were calculated based on effective dose coefficients derived from the International Commission on Radiological Protection, Publication 67 [22], wherein the amount of Cs activity detected by the WBC examinations is assumed to be in an equilibrium state between consecutive ingestions and excretions over the course of one year.

### Ethics

The present study was approved by the institutional review board of Jyoban Hospital, and written informed consent was obtained from all subjects enrolled in the study. For the children and minors, written informed consent was provided from the parents, relatives or caretakers.

## Results

**Fig 2:**
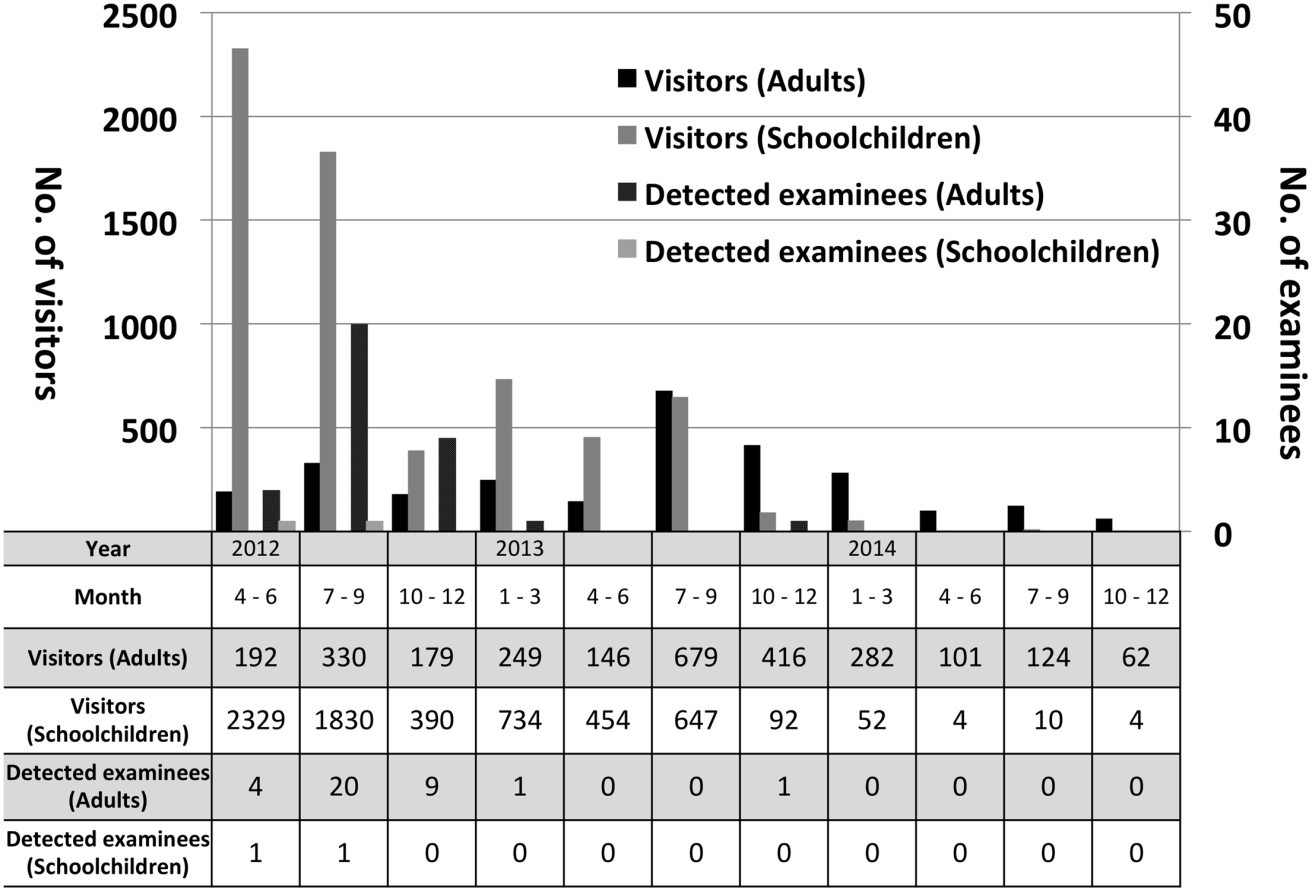
Monthly visitors and detected examinees.

Using WBC, internal radiation exposure screening was performed for Jyoban Hospital visitors between April 2012 and December 2014. A total of 9,206 subjects, including 6,446 schoolchildren, were enrolled (Fig 2). The children’s ages ranged from 4–15 years, and the median age was 5 years. Meanwhile, for adults ≥ 16 years, the median age was 48 years and the range was 16 to 95 years. Women accounted for 4,542 (49.2%) of the enrolled subjects.

### Annual effective dose from internal radiation exposure

**Fig 3:**
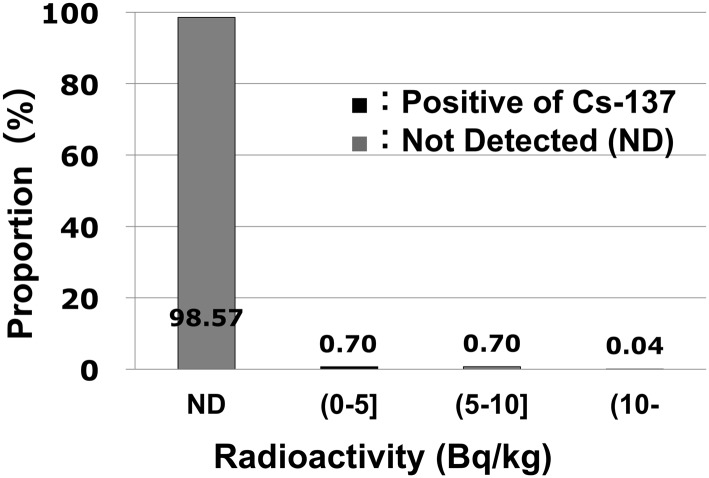
The proportion of doses attributed to internal Cs-137 radio-contamination in schoolchildren. Two children among the 6,446 children examined showed Cs-137 contamination levels that were slightly over the detection limit, although their Cs-134 levels were below the detection limit.

Among the 6,446 children tested, only two boys (11 and 15 years old) showed internal radio-contamination with Cs (Fig 3). The detected Cs-137 levels were just over the detection limit: 251 and 259 Bq/body, which correspond to doses of 0.018 and 0.011 mSv/year, respectively. Meanwhile, their Cs-134 levels were below the detection limit (220 Bq/body). The remaining 6,444 schoolchildren (99.9%) registered no internal Cs contamination that was within the detection limit of the WBC machines, and no additional radionuclides other than potassium-40 were seen in this screening. Assuming that the contamination levels in the children were just at the detection limit, the maximum annual effective doses from Cs-137 could be theoretically calculated as 0.03, 0.02, and 0.01 mSv/year for children aged 6, 10, and 15 years, respectively. These calculations were based on conservative assumption since the contamination levels in the most children without detection were considered far less than those of the detection limit of WBC machines. These findings suggest that the internal Cs contamination in schoolchildren living in areas south of the nuclear plant is minimal. These findings also support that programs to encourage consumption of only inspected foods and/or commercially distributed food from unaffected areas has been successful in controlling radio-contamination.

**Fig 4:**
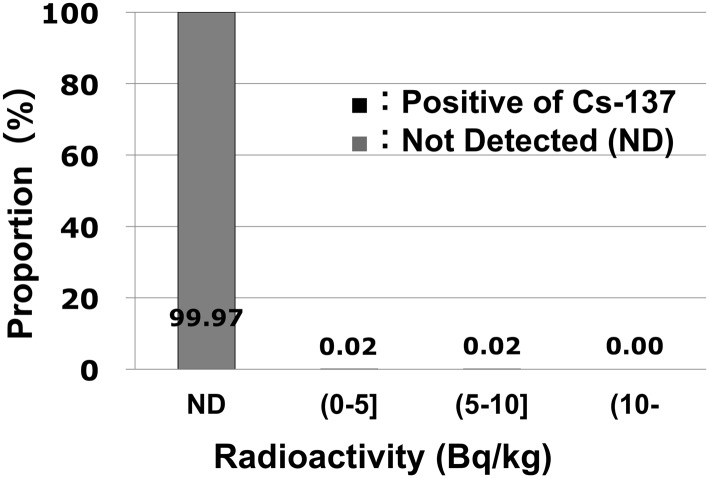
The proportion of doses attributed to internal Cs-137 radio-contamination in adults. Among the 2,760 adults examined, 35 (1.26%) had very low levels of Cs-137 contamination. Meanwhile, their Cs-134 levels were below the detection limit.

In sharp contrast to the study schoolchildren, 1.26% of the adults in the study (35/2760) had detectable levels of internal Cs contamination (Fig 4). Although the contamination measured in the two school children was detected only during the early stages of the screening program, detectable radio-contamination was seen in adults throughout the screening period, which ended in November 2014. The Cs-137 levels ranged from 250 to 859 Bq/body, with a median value of 318 Bq/body. These values correspond to doses of 0.008 to 0.028 mSv/year, respectively, with a median value of 0.011 mSv/year. No other radionuclides besides potassium-40 were detected for 98.7% of the examinees.

## Discussion

In the present study, 37 of the 9,206 study subjects (0.4%) showed detectable levels of internal Cs contamination. With the exception of one examinee who was evaluated in November 2014, by the end of 2013 (within 3 years of the incident), no other examinees displayed internal Cs contamination using the WBC monitoring method. Among the 37 subjects who did show Cs radio-contamination, the levels were minimal. Combined with a recent report that assessed 2,839 residents living in Iwaki city who showed committed effective doses ranging from 0.01 to 0.06 mSv/year [21], the situation for internal Cs contamination in the southern areas is similar to the northern and western areas, where the radiation exposure was far below the governmental limit (1 mSv/year) for the annual additional effective dose from internal radiation exposure.

Consistent with the findings presented here, previous studies reported that internal Cs contamination could be detected for nearly one year after the incident, and after this period very few subjects had detectable contamination [23]. We presume that the detection of internal Cs contamination during the early study stages was due to Cs that was derived from consumption of contaminated food and water, particularly before the systematic radio-inspection of locally grown products was established to control radionuclide uptake. During these early periods, the distribution system of safe, commercially available food was severely affected, since most employees of commercial food distribution companies within a 50 km radius of the crippled nuclear plant were forced to evacuate for several months, while residents within a 30–50 km radius remained in the affected areas. Thus, these residents were more likely to have eaten uninspected local food that may have had radio-contamination.

Radionuclide inhalation could also be a source of the internal Cs contamination detected in this screening. However, given the half-life of Cs in the body (3 months) and air doses in the affected areas, and that WBC screening began one year after the incident, inhalation is an unlikely cause of internal radio-contamination. We therefore presume that most of the contamination in the study subjects occurred during the early post-incident period when residents continued to consume locally grown products.

A notable limitation of the present study is that the WBC screening began one year after the incident, and no measurements were taken during the first year. As with other areas neighboring the affected nuclear plant, a mass of radionuclides was spread across southern areas, such that residents in those areas inhaled Cs intake and other highly radioactive compounds. In particular, immediately after the incident and up until the time when these residents were evacuated to areas such as Iwaki city, where contamination levels were far below the government-recommended limit of 1 mSv/year, these residents would have been exposed to high levels of contaminating radionuclides in the environment. In terms of health risk assessments, measurement of internal and external radiation exposure of residents living in areas south of the plant during the first year after the incident was critical. However, due to a lack of systemic measurement of exposure levels, especially during the first week after the incident, data for internal and external exposure of the residents are unavailable. As such, an accurate assessment of the health risks arising from radiation exposure is difficult, and the findings presented here can only partially describe the pattern of internal radio-contamination for residents living in areas to the south of the plant.

A WBC screening program to determine internal exposure was initially performed with residents living north (Minamisoma city) and west of the crippled nuclear plant [16]. Shortly after the incident, more than half of the examinees, including schoolchildren, showed internal Cs contamination, although the detected Cs levels in the schoolchildren were low and within safe limits (1 mSv/year) [17]. Over the course of one year, internal Cs contamination for the schoolchildren rapidly disappeared [18], and even for adults in the study detection of internal Cs contamination was quite rare [20]. Even though the present WBC screening started more than one year after the incident, the present findings for residents in areas to the south of the plant, together with previous findings for northern and western regions, now provide evidence that the affected areas surrounding the Fukushima Daiichi nuclear power plant are manageable in terms of internal Cs contamination. This situation is in contrast to residents affected by the Chernobyl disaster, who still showed high levels of internal Cs contamination more than one decade after the incident [24].

Consistent with the previous studies, the air dose in the affected areas likely does not reflect the internal radio-contamination assessed in the present study [19]. More recently, results from personal air dose equivalents estimated by glass badges revealed that both life style management and decontamination of residential areas were successful in protecting residents from radiation exposure in highly contaminated areas [25]. Taken together, undesirable radiation exposure appears to be generally controllable provided that affected residents pay close attention to conditions that may increase radiation exposure.

To avoid/reduce any chance of further internal Cs contamination, several strategies learned from previous incidents could be implemented. First, even if internal Cs contamination is detected, dietary interventions that promote consumption of inspected locally grown foods and commercially distributed food produced in unaffected areas appear to be effective in reducing contamination. For this purpose, continued WBC screening of affected residents and inspection of locally grown products is recommended. The importance of these efforts was verified by the long-term observations in areas affected by the Chernobyl incident [26]. Second, timely implementation of education programs to enhance residents’ awareness of radio-contamination risks is needed. Unfortunately, such efforts have not been tested since there are few scientists in the areas who could convey accurate but locally acceptable explanations of how residents can distinguish contamination due to released radionuclides from naturally radioactive potassium that is plentiful in many diets. Increased efforts such as informational lectures by local schoolteachers might be a promising approach to increase knowledge of radio-contamination risks. Likewise, should future nuclear plant malfunctions occur, public announcements of results from ongoing monitoring of residents’ internal radio-contamination levels could be an important incentive to help residents avoid the negative effects of radio-contamination.

On the other hand, excessive concerns about produce grown in affected areas may be detrimental to residents’ well-being, since such fears can discourage residents from engaging in outdoor activities, which may lead to decreased physical activity and an accompanying increase in the risk for non-communicable diseases [27]. Even under the assumption that the radio-contamination was at the highest level measured in the present study and that average lifestyles would maintain constant levels of Cs accumulation, the putative annual estimated sum of the internal exposure dose did not exceed the governmentally regulated limit dose of internal radiation exposure (1 mSv/year), but instead was less than 0.1 mSv/year. Although dietary modifications were successful in significantly reducing the internal Cs accumulation within six months, this lifestyle shift may be coupled with decreases in outdoor physical activity associated with harvests, and may impose a mental burden on affected residents who may be wary of produce grown locally in both gardens and fields. However, whether health risks such as increased tumor incidence are in fact due to radiation exposure remains unclear. Thus, dietary interventions that include avoiding food grown in the affected areas should be recommended to the extent that such limitations do not affect health and quality of life, particularly for elders, but instead should be implemented to a degree that they prevent incidence and development of non-communicable disease. In this respect, we must balance the health risk from reductions in the quality of life and threats imposed by internal radio-contamination.

## Conclusion

Internal radio-contamination levels in residents living in affected areas south of the nuclear plant disaster assessed from one year after the incident were minimal and generally well controlled. Since the annual effective dose derived from internal Cs contamination was negligible, nearly all of the annual effective doses in residents of southern areas more than one year after the incident might be due to external radiation exposure.
